# EVALUATION OF EDUCATION INITIATIVES TO INCREASE DELIVERY OF AGE-FRIENDLY CARE IN CONVENIENT CARE CLINICS

**DOI:** 10.1093/geroni/igad104.1860

**Published:** 2023-12-21

**Authors:** Nicholas Schiltz, Anna Bender, Grace Armstrong, Megan Foradori, Evelyn Duffy, Anne Pohnert, Mary Dolansky

**Affiliations:** Case Western Reserve University, Cleveland, Ohio, United States; University of Washingotn, Seattle, Washington, United States; Case Western Reserve University, Cleveland, Ohio, United States; Case Western Reserve University, Cleveland, Ohio, United States; Case Western Reserve University, Cleveland, Ohio, United States; CVS Health, Bellingham, Massachusetts, United States; Case Western Reserve University, Cleveland, Ohio, United States

## Abstract

The Age-Friendly Health Systems model of care reliably provides four evidence-based elements of high-quality care, to all older adult patients: What Matters, Medications, Mentation, and Mobility (the 4Ms). MinuteClinic at CVS implemented the 4Ms model in all 1100+ locations nationwide in May 2020. To prepare MinuteClinic providers to deliver 4Ms care, educational modules were developed to provide an understanding of the gerontology that supports the 4Ms model of care: an orientation module with two scenarios illustrating the usual care of an older adult patient followed by the same patient receiving 4Ms care, monthly grand rounds (case studies of older adult conditions with the 4Ms), and ten video vignettes regarding integration of 4Ms care. Our goal was to evaluate the effectiveness of these education initiatives, as measured by changes in the mean number of Ms delivered per visit. Over 70% of 3,188 providers completed at least one education module. The rate of 4Ms delivery was 1.7 times higher (p< 0.001) among those completing at least one education module, and 5.7 times higher (p< 0.001) among those completing ten or more modules compared to those who completed none. Each course was associated with significant within subject increases between 10% and 38% in 4Ms delivery in the month following course completion. The self-directed learning environment (e.g., providers self-select into number and type of courses) reflects real-world variation in engagement; despite this variation, improvements in 4Ms delivery were observed at any educational dose, underscoring the value of prioritizing education time with quality improvement initiatives.

